# STING signaling and host defense against microbial infection

**DOI:** 10.1038/s12276-019-0333-0

**Published:** 2019-12-11

**Authors:** Jeonghyun Ahn, Glen N. Barber

**Affiliations:** Department of Cell Biology, University of Miami Miller School of Medicine, Miami, FL USA

**Keywords:** Immunology, Innate immunity

## Abstract

The first line of host defense against infectious agents involves activation of innate immune signaling pathways that recognize specific pathogen-associated molecular patterns (PAMPs). Key triggers of innate immune signaling are now known to include microbial-specific nucleic acid, which is rapidly detected in the cytosol of the cell. For example, RIG-I-like receptors (RLRs) have evolved to detect viral RNA species and to activate the production of host defense molecules and cytokines that stimulate adaptive immune responses. In addition, host defense countermeasures, including the production of type I interferons (IFNs), can also be triggered by microbial DNA from bacteria, viruses and perhaps parasites and are regulated by the cytosolic sensor, stimulator of interferon genes (STING). STING-dependent signaling is initiated by cyclic dinucleotides (CDNs) generated by intracellular bacteria following infection. CDNs can also be synthesized by a cellular synthase, cGAS, following interaction with invasive cytosolic self-DNA or microbial DNA species. The importance of STING signaling in host defense is evident since numerous pathogens have developed strategies to prevent STING function. Here, we review the relevance of STING-controlled innate immune signaling in host defense against pathogen invasion, including microbial endeavors to subvert this critical process.

## Immunity: Fighting infection with a STING

In response to microbial infections a protein sensor named stimulator of interferon genes (STING) initiates the production of small defensive proteins called interferons. This is an early and ‘innate’ immune response, that is, one not targeted at specific invaders. Jeonghyun Ahn and Glen Barber at the University of Miami, USA, review the relevance of STING signaling in defense against infection, including consideration of microbial activities that can help the microbes evade this immune response. STING signaling is initiated by the presence of fragments of microbial genetic material called cyclic dinucleotides. These can be derived via cyclic GMP-AMP synthase (cGAS) from the DNA of invading viruses, bacteria or larger parasites such as single-cell protozoans. Discoveries revealing the significance and mechanism of our STING signaling system could lead to new strategies for combating infections, using either drugs or vaccines.

## Introduction

The innate immune system comprises the foremost line of host defense to counter invasive microbial agents^1,2^. Over the past two decades, host pattern-recognition receptors (PRRs) have been shown to play a key role in recognizing non-self, pathogen-associated molecular patterns (PAMPs). A variety of PRRs have now been reported, including the Toll-like receptors (TLRs), nucleotide-binding oligomerization domain (NOD)-like receptors (NLRs) and RIG-I-like receptors (RLRs)^1^. TLRs recognize extracellular or endosomal PAMPs, such as lipopolysaccharide (LPS), flagellin, single-stranded RNA, double-stranded RNA, and CpG DNA, to activate signaling through NF-κB, interferon regulatory factor (IRF) and MAP kinase signaling pathways, which induce cytokine production^2^. NLRs can also recognize PAMPs as well as damage-associated molecular patterns (DAMPs), including uric acid released by damaged cells, which trigger proinflammatory cytokine production^3^. The RLRs specifically recognize viral RNA species and activate analogous transcription factors and corresponding host defense-related molecules^1^. In addition, it is known that the presence of cytosolic DNA species can similarly trigger cytokine production^4^. This activity occurs because the cytosol is generally a DNA-free zone, and the existence of such nucleic acids usually signifies the arrival of an invading intracellular microbe or even leaked self-DNA from the nucleus as a result of DNA damage events. Cytosolic dsDNA species, generally over 70 bp in length, are now known to activate a host cyclic GMP-AMP synthase (cGAS), which generates cyclic dinucleotides (CDNs). These molecules bind, in turn, to an endoplasmic reticulum (ER)-associated sensor referred to as stimulator of interferon genes (STING), which results in NF-κB- and IRF3-dependent cytokine production^4–7^. Intracellular bacteria are also known to produce and secrete CDNs that directly activate STING signaling. Indeed, numerous DNA microbes have now been implicated in inadvertently triggering STING-dependent innate immune signaling and inducing cytokine production, including that of type I interferon (IFN)^4,7^. In response, there is growing evidence to indicate that a variety of microorganisms have attempted to evolve strategies to inhibit STING-dependent signaling. Here, we review the importance of STING-controlled innate immunity in preventing microbial infection, emphasizing how some of these pathogens try to subvert this critical host defense process. Understanding such host-pathogen interactions has important implications in the development of new therapeutic strategies to combat infectious disease.

### Activation of STING signaling

**Fig. 1:**
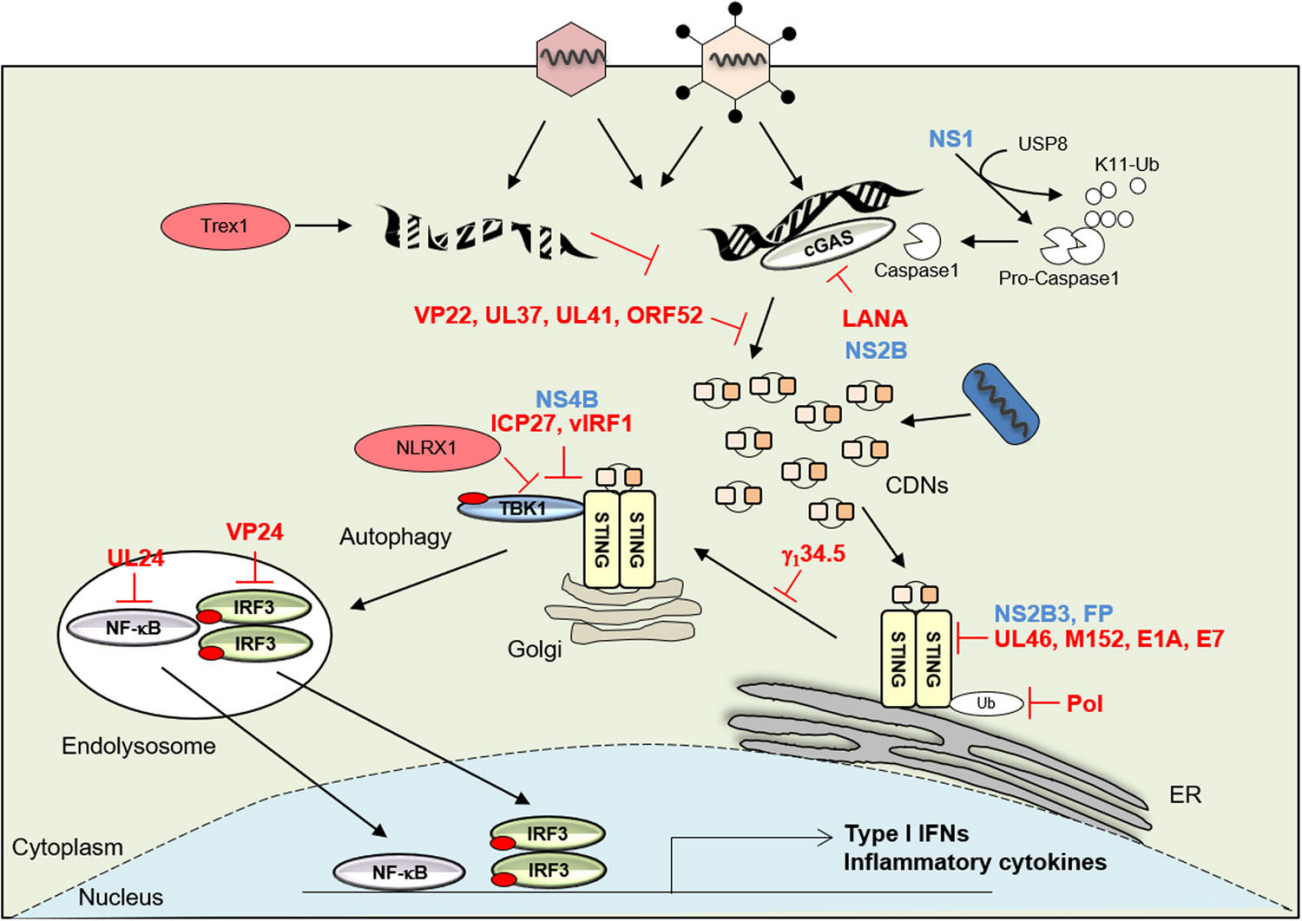
Activation of STING signaling and viral evasion. STING is activated by cyclic dinucleotides (CDNs) secreted by intracellular bacteria or non-canonical CDNs generated by cGAS. The sensing and interaction of CDNs influences a conformational change in STING and triggers the trafficking of STING complexed with TBK1 from the ER to endosomal/lysosomal perinuclear regions. Translocated TBK1 leads to the phosphorylation of IRF3 and NF-kB to induce type I IFNs or inflammatory cytokines. Microbial DNA or RNA interacts with cGAS/STING to evade critical innate immune signaling. Red letters: DNA virus proteins, blue letters: RNA virus proteins

The sensor STING was discovered following high-throughput screening of cellular molecules that could activate the IFNβ promoter^4,5^. STING, also known as transmembrane protein 173 (TMEM173), is a 379 or 378 amino acid protein in human or mouse cells, respectively^4,5,8–10^. Under normal conditions, STING is localized in the ER and is expressed mainly in hematopoietic cells, including macrophages, dendritic cells, natural killer cells, and T cells, as well as in endothelial and epithelial cells, which might be exposed to the environment and thus susceptible to infectious agents^4,5^. STING is a sensor that is activated by CDNs, such as cyclic-di-AMP, cyclic-di-GMP, and cyclic-GMP-AMP (3′3′-cGAMP; cyclic[G(3′,5′)]pA(3′,5′)p), secreted by intracellular bacteria, such as *Listeria monocytogenes*, or by non-canonical cyclic-GMP-AMP (2′3′-cGAMP; cyclic[G(2′,5′)]pA(3′,5′)p)) generated by cGAS^11–17^. The sensing and interaction of CDNs induces a conformational change in STING and triggers the trafficking of STING complexed with TANK-binding kinase 1 (TBK1) from the ER to endosomal/lysosomal perinuclear regions^4,5,18^. This event mimics a form of autophagy^4,19^. Translocated TBK1 leads to phosphorylation of the transcription factors interferon regulatory factor 3 (IRF3) and nuclear factor-κB (NF-κB), which translocate to the nucleus and initiate innate immune gene transcription^20,21^ (Fig. 1). Following these events, STING activity is suppressed, and then STING is rapidly degraded to avoid sustained cytokine production, which could lead to autoinflammatory disease^20^.

It is now well documented that STING plays an essential role in inducing type I IFN in response to sequence-nonspecific cytosolic DNA species that are greater than ~70 bp in human cells^4,5^. The requirement for large dsDNA species may be because cGAS needs to be in a dimeric form to be active, an event that requires two molecules of dsDNA, perhaps folded on themselves. Such DNA can constitute dsDNA oligonucleotides, single-stranded DNA forming hairpin duplexes, plasmids, and viral-, bacterial- or parasite-related DNA^4,5^. *Sting* knockout mice show high mortality following HSV-1 infection compared to that of wild-type mice^4^. STING has also been shown to be essential for the production of type I IFN induced by cytomegalovirus (CMV), vaccinia virus (VVΔE3L) and baculoviruses^4^. In addition, intracellular bacteria, such as *Listeria monocytogenes* and many others, may directly secrete STING-activating CDNs^22,23^. STING is not involved in dsRNA signaling, such as that by poly(I:C), which is largely governed by RLRs^4^. Nevertheless, loss of STING renders mice more susceptible to infection by select RNA viruses, such as vesicular stomatitis (VSV), suggesting that STING may play an important role in maintaining immune homeostasis^4,5,24^. Collectively, transient STING signaling plays a key role in protecting the host against a wide variety of pathogens, as described in more detail below. However, chronic STING activity may play a role in the development of autoinflammatory disease, underscoring the importance of tightly controlling this key innate immune signaling pathway^25,26^. This phenomenon may suggest that inflammatory events arising as a consequence of chronic infection may also involve the STING pathway, although this possibility remains to be clarified^25^.

### DNA virus activation and evasion of STING-dependent innate immunity

A variety of DNA viruses have been reported to activate STING signaling^4,5^. The mechanisms remain unclear, but the majority of these viruses inject their genomes from their protective capsids into the nucleus when they reach the nuclear pore^4,6,27,28^. Thus, microbial DNA may be exposed and susceptible to interactions with cGAS/STING. STING or cGAS knockout mice, as well as isolated macrophages and dendritic cells from those mice, have been shown to be susceptible to herpes simplex virus 1 (HSV1) and other DNA viruses^4,6^. However, while such agents may inadvertently activate STING, many viruses have developed strategies to suppress STING signaling to survive. For example, a slew of HSV-encoded products, including ICP27, γ34.5, UL24, UL36, UL37, UL41, UL42, VP11/12, VP22, and VP24, have been reported to abrogate cGAS/STING-mediated signaling (Fig. 1 and Table 1)^29–37^. HSV encodes a large dsDNA genome of ~150,000 bp and predominantly remains in latency in peripheral neurons^38^. In one case, Christensen et al. showed that ICP27 translocated to the cytoplasm, where it interacted with TBK1 and STING and inhibited IRF3 activation^29^. HSV-1 γ34.5 has also been reported to inactivate STING through disrupting the trafficking of STING from the endoplasmic reticulum to the Golgi apparatus^36^. HSV 1 serine proteases VP22 and VP24 have been shown to selectively block STING agonist-induced phosphorylation and dimerization of IRF3 but not NF-κB activation^30,31^. VP22 also interacted with cGAS to inhibit its enzymatic activity^31^. UL24 was shown to prevent cGAS/STING-mediated IFNβ and interleukin-6 (IL-6) production by selectively blocking nuclear factor-κB (NF-κB) but not IFN-regulatory factor 3 function^35^. One of the most abundant HSV tegument proteins, UL46, was demonstrated to interact with STING to prevent activity^32^. It has also been reported that an additional tegument protein, UL41, reduced the accumulation of cGAS, which prevented CDN production^33^, and UL37 deamidated cGAS, similarly resulting in impaired CDN production^34^. Finally, HSV-1 ubiquitin-specific protease (UL36USP) antagonizes NF-kB activation induced by the STING pathway^37^. It is unclear why HSV may encode so many apparent ways to prevent STING signaling, but suppressing this pathway must be important for its survival. Perhaps this herpesvirus member utilizes varying suppressive methods at different stages of its life cycle, from entry to latency to its lytic phase.

**Table 1:** DNA virus evasion of STING-dependent innate immunity

Virus	Viral genes	Mechanisms	References
HSV-1	ICP27	Interacts with TBK1 and STING and prevent IRF3 activation.	^29^
	g_1_34.5	Disrupts STING trafficking from ER to Golgi and inhibit IRF3 activation.	^36^
	VP22	Interact with cGAS and Inhibit the enzymatic activity of cGAS.	^31^
	VP24	Blocks phosphorylation and dimerization of IRF3 but not NF-kB.	^30^
	UL24	Prevents IL-6 production by NF-kB.	^35^
	UL46	Colocalizes with STING and inhibit interferon stimulating gene transcription.	^32^
	UL41	Decreases cGAS accumulation and prevent cGAMP production.	^33^
	UL37	Deamidates cGAS, similarly resulting in impaired CDN production.	^34^
	UL36	Block promoter activation IFNb and NF-kB induced by cGAS and STING depending on its deubiquitinase activity.	^37^
KSHV	vIRF1	Prevents STING from interacting with TBK1.	^40^
	ORF52	Binds to both DNA agonist and cGAS and impede CDN production.	^41^
	LANA	Directly binds to cGAS and inhibit STING signaling.	^42^
CMV	M152	Binds to STING and inhibit STING signaling.	^45^
HBV	Pol	Interferes with the K63-linked polyubiquitination of STING via its reverse transcriptase (RT) domain.	^50^
Ad	E1A	Inhibits the cGAS/STING pathway by directly binding to STING.	^28^
HPV	E7	Inhibits the cGAS/STING pathway by directly binding to STING.	^28^

However, another member of the herpesvirus family, Kaposi sarcoma herpes virus (KSHV), known as human herpesvirus 8 (HHV-8), is similarly a large double-stranded DNA virus able to trigger STING activity, which causes Kaposi’s sarcoma (KS)^39^. However, Ma et al. reported that KSHV-encoded vIRF1 inhibited this pathway by preventing STING from interacting with TBK1^40^. Wu et al. additionally reported that KSHV ORF52, an abundant gamma herpesvirus-specific tegument protein, may impede CDN production through binding to both the DNA agonist and cGAS^41^. Furthermore, latency-associated nuclear antigen (LANA) of KSHV may inhibit STING signaling by directly binding to cGAS. This effect could conceivably antagonize cGAS-mediated restriction of KSHV’s lytic replication^42^. It remains to be seen whether other members of the herpesvirus family inhibit STING signaling. For example, CMV has been reported to trigger STING signaling following infection^43,44^. At least in mice, murine CMV (MCMV) may encode a product referred to as M152, which binds to STING to suppress this response^45^. Varicella zoster virus (VZV/HHV3) has also been documented to trigger STING signaling, although direct suppression of signaling has not yet been reported. It should be noted, however, that many of these and other viruses have also been shown to inhibit interferon signaling downstream of STING at the level of IRF3 or Jak/STAT signaling, indicating that suppression of host defense responses occurs at many levels^46–48^.

Hepatitis B virus (HBV), containing a circular DNA genome, specifically infects hepatocytes and causes chronic hepatitis^49^. Evidence indicates that HBV can decrease IFNβ production in transiently HBV-transfected Huh7 cells; stably HBV-producing cell lines, such as HepAD38; HBV-infected HepaRG cells; and primary human hepatocytes. The viral polymerase (Pol) of HBV has been reported to interfere with K63-linked polyubiquitination of STING via its reverse transcriptase (RT) domain^50^. However, it is still controversial whether HBV infection elicits a detectable cytokine response in hepatocytes, at least through STING. While one group reported that human hepatoma cells as well as immortalized mouse hepatocytes express low levels of STING^51,52^, another group indicated that human and murine hepatocytes do not express STING and do not produce type I IFN in response to foreign DNA or HBV infection^51^. Indeed, it is tempting to speculate that some viruses may target cells that may lack certain innate immune sensing pathways. Nevertheless, Kupffer cells, as stellate macrophages located in the liver, may express STING, and contribute toward the clearance of dying infected hepatocytes to possibly influence inflammation.

Other double-stranded DNA viruses, such as adenovirus (Ad) and human papillomavirus (HPV), have similarly been shown to antagonize the cGAS/STING DNA-sensing pathway^27,28,53^. Following Ad infection, cells deficient in STING or cGAS expression were noted to lack IRF3 phosphorylation, and activation of IFNβ or IRF3-responsive genes, such as ISG15 and ISG54, was compromised^27,53^. The oncogene E1A from Ad and E7 from HPV reportedly inhibit the cGAS/STING pathway by directly binding to STING. Suppression of E1A and E7 expression could restore the production of type I IFNs^28^. Finally, Eaglesham et al. showed that the large cytosolic DNA virus, vaccinia virus similarly suppresses STING via the production of poxins which cleave CDNs. Collectively, it is perhaps unsurprising that DNA viruses have evolved mechanisms to suppress dsDNA-triggered innate immune signaling. Many of the viruses noted here can remain latent and even contribute toward tumorigenesis. It is unclear whether suppression of STING signaling may influence the transformation process. Evidence now indicates that STING signaling is suppressed in many types of tumor cells, presumably to avoid DNA damage-activated immune responses^54,55^. In addition, STING activity has been shown to be important for the generation of antiviral as well as antitumor T cells. Thus, suppression of cGAS/STING not only may help DNA viruses survive but also may contribute toward cellular transformation.

### The STING signaling pathway and retroviral infection

Host defense gene induction has been reported to also occur following retrovirus/lentivirus entry^56^. Following infection, the viral single-stranded RNA genome is reverse transcribed and delivered to the nucleus via mature integration complexes. STING has been reported to colocalize with such complexes^57^. Perhaps as a result, cGAS/STING knockout mice are defective in HIV-, murine leukemia virus-, and simian immunodeficiency virus-triggered type I IFN production^7^. However, the production of type I IFN is generally weak. This phenomenon may be due to agonist viral DNA species in the cytosol being degraded by cytoplasmic DNases, such as Trex1, a 3′–5′ exonuclease^58^. In the absence of Trex1, genomic or viral DNA accumulates in the cytosol and activates STING-dependent innate immune signaling^59^. In humans, mutations in Trex1 cause inflammatory diseases, such as Aicardi-Goutieres syndrome (AGS) and severe systemic lupus erythematosus (SLE)^26,60,61^. In experimental conditions, Trex1 deficiency reportedly results in increased HIV replication and type I IFN production^61^. Moreover, two single nucleotide polymorphisms (SNPs) in Trex1 have been documented in humans as being associated with faster HIV-1 disease progression and increased HIV replication^62^. Another negative regulator of innate immunity, a member of the nucleotide-binding domain, leucine-rich repeat-containing proteins (NLRs), NLRX1, has also been described as associating with STING to reduce TBK1 activity and enable increased HIV-1 infection^63,64^. Human T lymphotropic virus type 1 (HTLV-1), a member of the delta retrovirus family, is the causative agent of adult T cell leukemia (ATL) and tropical spastic paraparesis (TSP)^65^. HTLV-1 reverse transcription intermediates (RTIs) have been shown to trigger STING-dependent IFNβ production in differentiated human macrophages, including THP1 cells. It has also been reported that HTLV-1 RTIs interact with STING and induce IRF3-Bax complexation, leading to apoptosis^66^. The HTLV-1 protein Tax has been shown to impair IFNβ production by influencing K63-linked ubiquitination of STING to disrupt interactions between STING and TANK-binding kinase 1 (TBK1)^67^. Thus, retroviruses/lentiviruses have evolved to avoid robust STING activation and may be assisted by molecules such as Trex1. It should be noted that up to 10% of the human genome contains versions of ancient retroviruses referred to as human endogenous retroviruses (ERVs)^68^. In addition, over 40% of the human genome consists of retrotransposons, which are DNA components that can be transcribed into RNA and converted back into identical DNA sequences by a reverse transcriptase encoded by the retrotransposon itself^68^. It is unclear whether such ERVs or retrotransposons aggravate innate immune signaling when reactivated to cause inflammatory disease^59,69,70^.

### RNA virus infection and STING-dependent innate immunity

As discussed, STING signaling controls CDN- and cytosolic DNA-triggered innate immune signaling. However, early studies quickly showed that STING knockout mice were also susceptible to RNA viruses, such as VSV^4^. Usually, these pathways are governed by the RLR pathway, TLR3 and TLR7^1,2^. However, type I IFN production was noted as being decreased in STING knockout cells infected with VSV. This result implies that STING is also necessary for protection against certain RNA viruses^4^. Recently, it was reported that STING may also restrict the replication of various RNA viruses at the posttranslational level^71^. This effect may be due to STING residing in the ER of the cell and being associated with the translocon, a portal where proteins destined for glycosylation and/or secretion are held for appropriate maturation^5,25^. The role of STING in translocon function remains to be clarified. Regardless, growing evidence now indicates that certain RNA viruses target STING for suppression (Fig. 1 and Table 2).

**Table 2:** Blocking STING-dependent innate immunity by RNA virus infection

Virus	Viral genes	Mechanisms	References
HCV	NS4B	Disrupts the interaction between STING and TBK.	^75,76^
DENV	NS2B3	Targets and cleavages wild type human STING to prevent type I IFN production.	^78,79,81^
	NS2B	Targets cGAS to prevent mitochondrial DNA sensing released during DENV infection.	^80^
ZIKV	NS2B3	Cleaves R78 and G79 in the cytoplasmic loop of human STING.	^83^
	NS1	Recruits the deubiquitinase USP8 to cleave K11-linked ubiquitin chains of caspase-1 and the caspase-1 targets to cGAS for cleavage.	^84^
IAV	FP	Interacts with STING to antagonize type I IFN production.	^89^

Hepatitis C virus (HCV) is an enveloped, positive-sense, single-stranded RNA virus in the family Flaviviridae that causes hepatitis and facilitates cancer development, such as that of hepatocellular carcinoma^72^. NS3/4A and NS4B, a serine protease of HCV, targets IPS1/MAVS/Cardif, a CARD-containing adaptor protein to block type I IFN production via RLRs^73^. In addition, STING-dependent IFNβ activation was observed to be suppressed by NS4B^74^. It is possible that NS4B disrupts the interaction between STING and TBK in STING- and TBK1-overexpressing cells transfected with the NS4B plasmid^75,76^. Similar to the situation with HBV infection, it is not clear whether STING is highly expressed in HCV-infected hepatocytes. However, Kupffer cells may play a key role in viral clearance and plausibly in inflammation associated with hepatitis-related diseases.

Dengue virus (DENV) is a mosquito-borne single-, positive-stranded RNA virus belonging to Flaviviridae that causes hemorrhagic fever in humans^77^. It has been documented that the DENV NS2B3 protease can inhibit type I IFN production through its proteolytic activity. It was shown that the protease of DENV targets and cleaves wildtype STING to prevent type I IFN production. DENV replication is highly increased in STING-deficient primary cells^78,79^. Recently, Aguirre et al. reported that NS2B also targets cGAS for degradation in an autophagy-lysosome-dependent mechanism to prevent sensing of mitochondrial DNA released during DENV infection^80^. Furthermore, the protease of dengue virus 2 (DENV2) cleaves human but not primate STING, reducing type I interferon production and boosting viral titers^81^. However, another positive-stranded RNA virus, which closely resembles DENV, is Zika virus (ZIKV), first isolated in Uganda in 1947. Recently, a large outbreak of malaise was identified as involving Zika infection in Brazil in 2015; thereafter, cases of outbreaks and evidence of transmission soon appeared worldwide, including in the Americas. It has been reported that different non-structural proteins of ZIKV, such as NS1 and NS4B, decrease the innate antiviral response to evade the host immune response^82^. Similar to DENV, the NS2B3 protease of ZIKV cleaves R78 and G79 in the cytoplasmic loop of human STING^83^. In an analysis of the host tropism of ZIKV, rodents, unlike humans, are not susceptible to ZIKV infection. This difference may be due to R78 and G79 being only partially conserved in the murine ortholog of STING^83^. In addition, Zheng et al. have shown that the NS1 protein of ZIKV recruits the deubiquitinase USP8 to cleave K11-linked ubiquitin chains at lysine 134 of caspase-1. Subsequently, caspase-1 targets cGAS for cleavage, which results in a reduction in type IFN production^84^. ZIKV is known to cause microcephaly in newborns, although the mechanisms and frequency of this syndrome remain to be clarified. One group has shown that STING-dependent signaling plays a role in antiviral macroautophagy/autophagy to restrict ZIKV infection in the fly brain. This study in *Drosophila* reveals key insights into the evolutionary function of STING in antiviral defense and further evidence for the ancestral function of autophagy in protecting host cells from viral invaders^85–87^.

Influenza A viruses (IAVs), in contrast, are negative-sense, single-stranded, segmented viruses that may suppress STING signaling^88^. In this regard, the hemagglutinin fusion peptide (FP) of IAV reportedly interacts with STING to antagonize type I IFN production in a STING-dependent but cGAS-independent manner^89^.

Thus, STING may also play an evolutionarily important role in protecting the host against microbial infection. In this light, it is worth noting that many RNA viruses, including DENV and ZIKV, are able to infect both human and insect cells. It is unclear whether such viruses suppress STING in their insect hosts if STING is expressed. Indeed, many viruses may only be able to succeed in hosts/cells where STING or similar innate immune pathways are absent.

### Bacteria, CDNs, and STING-dependent innate immunity

STING is a direct sensor of CDNs, including c-di-GMP and c-di-AMP, generated by numerous intracellular bacteria, such as *Listeria monocytogenes*^90^. CDNs play a significant role in the life cycle of such bacteria, functioning as second messengers^11^. *Listeria monocytogenes* (*L. monocytogenes*) infection reportedly induces type I IFN and IL6 in wild-type murine fibroblasts, macrophages, and dendritic cells and in vivo, which is dependent on STING via CDNs^4,23,91^. *L. monocytogenes* secretes c-di-AMP through multidrug efflux pumps (MEPs)^11^. Moreover, *L. monocytogenes* DNA is also able to stimulate the IFN response in the STING/cGAS pathway in human macrophages^22^. STING likely evolved to detect CDNs early in evolution. The synthase cGAS probably later evolved to generate CDNs following interaction with DNA. Thus, STING may have been predominantly involved in innate immunity to bacterial infection and even RNA virus infection (through its speculative translocon function) before becoming central in innate immune signaling pathways triggered by DNA^5^.

Extracellular pathogens, such as *Streptococcus pneumoniae* (*S. pneumoniae*), are some of the leading causes of death in people over the age of 65 years. *S. pneumoniae* has been known to induce type I IFN and to regulate RANTES production through STING^92,93^. STING-dependent type I IFN production in elderly mice was decreased following *S. pneumoniae* infection. *S. pneumoniae* infection induces ER stress and augments inositol-requiring protein 1/X-box binding protein 1-mediated production of autophagy-related gene 9 (Atg9a)^94^. Saito et al. showed that a loss of Atg9 enhances the assembly of STING/TBK1 and increases innate immune signaling^19^. This result indicates that Atg9 induction by ER stress could decrease STING activity by *S. pneumoniae* infection, providing new evidence as to why older people may be more susceptible to infection.

*Mycobacteria tuberculosis* (*M. tuberculosis*), the causative agent of tuberculosis, remains one of the leading causes of chronic infectious pulmonary disease^95^. *M. tuberculosis* activates a cytosolic surveillance pathway (CSP) and induces innate immune responses following perforation of the phagosome membrane. This effect is mediated by the microbe’s ESX-1 secretion system following interaction with target macrophages^96^. Permeabilization mediated by ESX-1 allows cytosolic components of the ubiquitin-mediated autophagy pathway access to *M. tuberculosis* in phagosomes. Consequently, the STING pathway recognizes the extracellular bacterial DNA and activates innate immune responses^96–99^. CDNs can also be generated by such microbes, which can directly activate STING^100,101^. Dey et al. reported that c-di-AMP produced by *M. tuberculosis* controls the fate of infection by stimulating IFNβ production, an event that may actually facilitate bacterial survival^100,101^.

In addition to the bacteria described here, various other microbes, such as *Chlamydia, Francisella, Brucella, Shigella, Salmonella*, and *Neisseria*, have been reported to engage the STING-dependent pathway^102^. However, while STING has likely evolved to recognize bacterial infection through recognition of the CDNs produced, the role of such CDNs in manipulating STING signaling, perhaps even to facilitate their survival, remains an interesting area of study, which will likely help explain mechanisms of pathogenesis^102^.

### Parasites, malaria and STING signaling

*Plasmodium* parasites cause malaria, a debilitating disease affecting millions worldwide. Malaria infection is initiated by mosquitos injecting infectious sporozoites following biting their host. These sporozoites are transferred to the liver via the bloodstream. After replication in the liver, infectious exoerythrocytic merozoites are released into the blood^103^. Miller et al. showed that plasmodiums in the liver induce type I interferon-mediated innate immune responses. Type I IFN activates NKT cells, which produce IFNγ to inhibit secondary liver-stage infection^104^. Malaria-specific parasites inside red blood cells secrete extracellular vesicles (EVs) containing parasitic small RNA and genomic DNA. Human monocytes can take up the EVs, and parasitic DNA is released into the host cell cytosol, where STING is activated^105^. However, it has also been shown that TLR7 in pDCs can also contribute to type I IFN production in response to malaria infection in a murine model^106^. Thus, STING signaling may contribute toward protection of the host against malaria. Whether STING also plays a crucial role in protecting the host against other types of parasites remains to be seen.

### Potential of STING in new antipathogen strategies

STING signaling plays an important role in stimulating the immune system in response to microbial infection, suggesting that control of this pathway may be useful in antimicrobial strategies to control disease. As described, various CDNs, such as cyclic-di-AMP, cyclic-di-GMP, and cGAMP (and synthetic analogues), can stimulate STING activity^12,14–16^. Indeed, STING agonists are now being evaluated in the clinic to enhance antitumor immunity^107–110^. Evidence indicates that the injection of CDNs into tumors stimulates surrounding antigen-presenting cells (APCs) to augment antitumor CTL activity^111,112^. Similarly, it is possible that comparable strategies may exert useful antimicrobial activity. In one example, reports indicate that systemic or local application of 2′3′-cGAMP reduces genital HSV-2 replication and improves the clinical outcome of infection, with strong induction of type I IFNs both in human cells and in mice in vivo^113^.

In addition to CDNs, alternate STING agonists have also been reported. For example, 5,6-dimethylxanthenone-4-acetic acid (DMXAA) and 10-(carboxymethyl)-9(10H) acridone (CMA) are flavonoids that potently bind to and activate STING signaling^114,115^. In a hepatitis B virus (HBV) hydrodynamic mouse model, DMXAA induced IFN-stimulated genes and decreased HBV DNA replication in the livers of mice. Since chronic HBV infection involves failure of the host to induce a sufficient immune response to clear the virus, such strategies indicate that activation of the STING pathway by agonists may be useful in treating such diseases^116^. In another example, a group identified novel IFN/IRF3-inducing molecules by high-throughput in vitro screening, referred to as 4-(2-chloro-6-fluorobenzyl)-*N*-(furan-2-ylmethyl)-3-oxo-3,4-dihydro-2H-benzo[b] thiazine-6-carboxamide (G10), and *N*-(methylcarbamoyl)-2-{[5-(4-methylphenyl)-1,3,4-oxadiazol-2-yl]sulfanyl}-2-phenylacetamide (C11)^117,118^. G10 reportedly induced IFN/IRF3-dependent signaling but not NFĸB signaling. This compound mediated anti alphaviral activity against chikungunya virus (CHIKV), Venezuelan equine encephalitis virus (VEEV) and Sindbis virus (SINV) and required STING- but not IPS-1/MAVS-dependent signaling^118^. C11 was also able to induce IFN secretion in human cells in a manner that required STING but not MAVS or TRIF. C11-treated cells potently blocked the replication of multiple emerging alphavirus types, including chikungunya, Ross River, Venezuelan equine encephalitis, Mayaro, and O’nyong’nyong viruses^117^. Thus, the use of STING agonists may be of benefit in treating microbial disease as well as in immune cancer therapy.

Finally, it is noteworthy that STING agonists may also be useful as vaccine adjuvants for the stimulation of the STING-dependent innate immune pathway. A number of examples now demonstrate the usefulness of such approaches in vaccine development to protect against microbes^107–110,116–118^. For example, CDN-formulated vaccines elicited long-lasting protective immunity against *Mycobacterium tuberculosis* in a murine model similar to that elicited by live attenuated vaccine strains presently in use, such as Bacille Calmette-Guérin (BCG)^119^.

The discovery of the STING signaling pathway has provided considerable insight into microbial pathogenesis, mechanisms of host defense, and causes of inflammatory disease and even cancer. These discoveries have led to investigation of whether controlling the STING pathway can generate new vaccines as well as antimicrobial agents to control a variety of diseases.
